# Structural Basis for the Function of the C-Terminal Proton Release Pathway in the Calcium Pump

**DOI:** 10.3390/ijms22073507

**Published:** 2021-03-29

**Authors:** L. Michel Espinoza-Fonseca

**Affiliations:** Center for Arrhythmia Research, Department of Internal Medicine, Division of Cardiovascular Medicine, University of Michigan, Ann Arbor, MI 48109, USA; lmef@umich.edu; Tel.: +1-734-998-7500

**Keywords:** SERCA, calcium, proton transport, molecular dynamics

## Abstract

The calcium pump (sarco/endoplasmic reticulum Ca^2+^-ATPase, SERCA) plays a major role in calcium homeostasis in muscle cells by clearing cytosolic Ca^2+^ during muscle relaxation. Active Ca^2+^ transport by SERCA involves the structural transition from a low-Ca^2+^ affinity E2 state toward a high-Ca^2+^ affinity E1 state of the pump. This structural transition is accompanied by the countertransport of protons to stabilize the negative charge and maintain the structural integrity of the transport sites and partially compensate for the positive charges of the two Ca^2+^ ions passing through the membrane. X-ray crystallography studies have suggested that a hydrated pore located at the C-terminal domain of SERCA serves as a conduit for proton countertransport, but the existence and function of this pathway have not yet been fully characterized. We used atomistic simulations to demonstrate that in the protonated E2 state and the absence of initially bound water molecules, the C-terminal pore becomes hydrated in the nanosecond timescale. Hydration of the C-terminal pore is accompanied by the formation of water wires that connect the transport sites with the cytosol. Water wires are known as ubiquitous proton-transport devices in biological systems, thus supporting the notion that the C-terminal domain serves as a conduit for proton release. Additional simulations showed that the release of a single proton from the transport sites induces bending of transmembrane helix M5 and the interaction between residues Arg762 and Ser915. These structural changes create a physical barrier against full hydration of the pore and prevent the formation of hydrogen-bonded water wires once proton transport has occurred through this pore. Together, these findings support the notion that the C-terminal proton release pathway is a functional element of SERCA and also provide a mechanistic model for its operation in the catalytic cycle of the pump.

## 1. Introduction

The calcium pump (sarco/endoplasmic reticulum (SR) Ca^2+^-ATPase, SERCA) is an intensely studied ATP-dependent transmembrane ion pump, truly the biophysical model for active transport and energy transduction [1] in more than 600 homologous P-type ion pumps [2]. SERCA actively transports Ca^2+^ from the cytosol back into the SR lumen of cells at the expense of ATP hydrolysis, thus playing vital roles in Ca^2+^ homeostasis and signaling [3]. SERCA-mediated Ca^2+^ transport across the SR membrane is accompanied by the concomitant flux of protons from the lumen to the cytosol to stabilize the negative charge and maintain the structural integrity of the transport sites [4] and to partially compensate for the positive charges of the two Ca^2+^ ions passing through the membrane [5]. Consequently, proton currents through SERCA play an essential role in balancing the charge deficit that occurs during active ion transport across the SR membrane.

**Figure 1:**
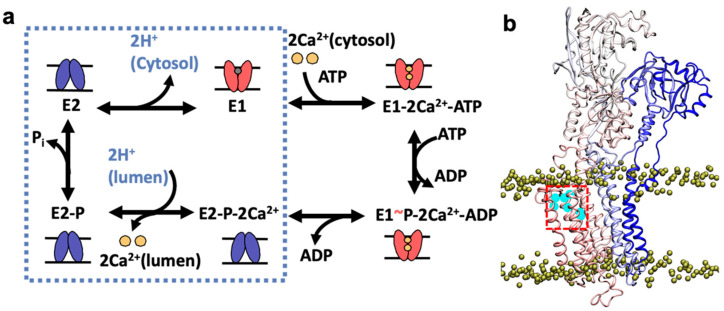
Location of the C-terminal proton release pathway, as suggested by x-ray crystallography studies. (**a**) Schematic representation of SERCA (sarco/endoplasmic reticulum (SR) Ca^2+^-ATPase) transport cycle showing the major biochemical intermediates of the pump. Proton transporting events in the cycle are shown in the dashed box. Low-Ca^2+^ affinity (E2) and high-Ca^2+^ affinity (E1) states of SERCA are shown in blue and red, respectively. (**b**) The pathway is shown inside the red box showing the presence of crystallographic water molecules inside this pore (cyan). We show the location of the N-terminal (blue) and C-terminal (red) regions of SERCA. The yellow spheres show the boundaries of the lipid bilayer. For this figure, we used the crystal of SERCA in the E2 state bound to AlF_4_^-^ and the inhibitor thapsigargin (PDB: 3n5k [18]).

SERCA undergoes major structural transitions that are required for Ca^2+^-mediated activation of the pump [6,7,8]. During each cycle, SERCA populates a high-Ca^2+^ affinity state (E1), that binds two Ca^2+^ ions from the cytosol to the transmembrane transport sites and one molecule of ATP in the nucleotide-binding domain. This nucleotide-bound E1-2Ca^2+^-ATP complex facilitates ATP utilization and the formation of the phosphorylated intermediate E1~P-2Ca^2+^-ADP [9]. SERCA then undergoes a structural transition toward a phosphorylated E2-P_i_ intermediate that has a low affinity for Ca^2+^, thus facilitating translocation of two Ca^2+^ ions from the transport sites into the SR lumen. Two protons from the lumen then bind to the transport sites to neutralize the negative charge in the TM domain and stabilize this intermediate state [4,10,11,12]. The pump then undergoes dephosphorylation [13,14] to form a protonated, low-Ca^2+^ affinity E2 state [6,7,9]. This E2 intermediate undergoes further deprotonation [10,15,16,17] to induce an E2-to-E1 transition of SERCA required to initiate a new catalytic cycle of the pump [6,7,8]. These transitions are summarized in Figure 1a.

During this last step in the cycle of the pump, protons are released from the transport sites to the cytosol primarily via two pathways: (i) from residue Glu309 through the N-terminal pathway to allow the opening of the cytosolic gate that is needed for binding of metal ions to the transport sites N-terminal [10,15,19], and (ii) through a C-terminal proton release pathway cytosolic pathway to allow proton translocation from SERCA residue Glu908 to the cytosol [18]. Proton release through the N-terminal pathway is a relatively straightforward event that involves the formation of a short, transient proton-release pathway connecting Glu309 with the cytosol [19]; the pore is then closed following the binding of two Ca^2+^ ions in the transport site [18]. The presence of a C-terminal pathway has remained elusive, and it has only inferred from X-ray crystallography studies showing the presence of a partially hydrated pore on the cytosolic side of the C-terminal region SERCA in the E2 state bound to AlF_4_^-^ and the inhibitor thapsigargin (Figure 1b) [18]. If this proton pathway exists, as suggested by crystallography studies, it would represent the missing functional component that explains proton countertransport during the catalytic cycle of the pump, as it provides an operational conduit for proton release during the exchange of metal ions and a proton in the transport sites during the E2-to-E1 transition. In this study, we used atomistic simulations to probe the functionality of the C-terminal proton release pathway at physiological-like conditions.

## 2. Results and Discussion

**Figure 2:**
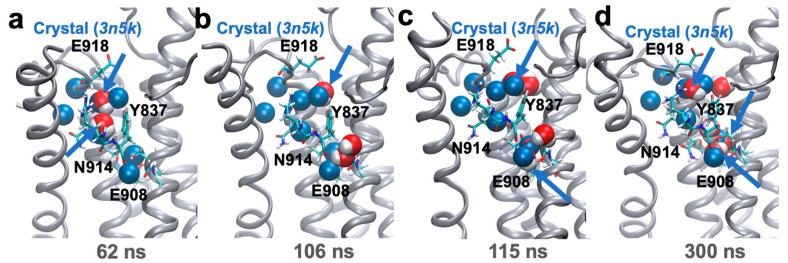
Hydration pattern of the C-terminal proton release pathway detected in the 300 ns simulation of the protonated E2 state of SERCA. (**a**) Water penetration into the pathway observed at t = 62 ns; (**b**) and (**c**) penetration of water molecules into the pore near transport site residue Glu908; (**d**) orientation of water molecules in the C-terminal pathway observed at the end of the trajectory. In all cases, SERCA is shown as ribbons, key residues along the C-terminal pathway as sticks, and water molecules as red/white spheres; for comparison, we show the location of the crystallographic water molecules (PDB: 3n5k [18]) as blue spheres. The blue arrows indicate the location of water molecules in the simulation that overlap (RMSD < 2Å) with the crystallographic waters.

We first point out that the crystal structure that more closely characterizes the ‘native’ E2 state of SERCA, i.e., determined in the absence of non-cognate ligands (PDB: 3w5c, ref. [20]) does not contain water molecules in the C-terminal proton release pathway. Therefore, we first performed a single 300 ns molecular dynamics (MD) simulation starting from this crystal structure in an explicit lipid and water environment to (i) to demonstrate that the C-terminal of the E2 state is intrinsically occupied by water molecules, as suggested in earlier studies [18], and (ii) to generate an unbiased structure with the characteristics of the partially hydrated C-terminal pore of the protonated E2 state of SERCA. The position of the residues along the proton pathway is virtually unchanged in the nanosecond timescale used here, as revealed by the root mean square deviation (RMSD) analysis of residues Ser767 (0.9 ± 0.1 Å), Arg836 (1.1 ± 0.3 Å), Tyr837 (0.8 ± 0.3 Å), Asn911 (1.3 ± 0.3 Å), and Asn914 (0.9 ± 0.1 Å). These results indicate that the structural stability of the C-terminal pathway is not affected by the absence of initially bound water molecules in this region of the protein. We found that two water molecules from the cytosolic side of the complex bind to the pore at t = 62 ns to a cavity formed by residues Tyr837, Asn914, and Glu918 (Figure 2a). Residue Tyr837 then recruits two water molecules from the cytosol at t = 106 ns (Figure 2b), and further recruited by residue Glu908 at t = 115 ns (Figure 2c). At the end of the trajectory, five water molecules occupy the C-terminal pore (Figure 2d). Remarkably, the positions of three water molecules in the pore overlaps remarkably well with those found in the crystal structure of the E2 state bound to AlF_4_^-^ and thapsigargin (Figure 2d). The qualitative correspondence between simulations and experimental data confirms the crystallography-based hypothesis that the binding of water molecules in this pore is an intrinsic feature of the protonated E2 state of the pump [18].

**Figure 3:**
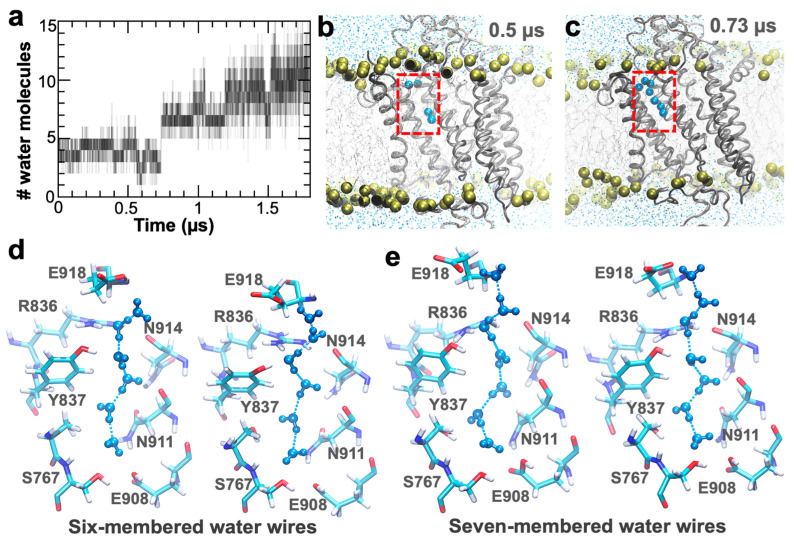
Identification of water wire formation in the C-terminal pathway of the protonated E2 state of SERCA. (**a**) The number of water molecules present in the C-terminal pore throughout the 1.8 µs of simulation time. The number of water molecules was computed at 1 ns intervals. (**b**) and (**c**) show the configuration of the water molecules in the partially and fully hydrated structures of the C-terminal pathway. The location of the pore is shown inside the red box; water molecules in the pore are shown as blue spheres, SERCA as ribbons, the lipid bilayer as yellow spheres and sticks, and bulk water in the cytosolic and luminal sides as blue dots. (**d**) and (**e**) Structure of the six-membered and seven-membered water wires that connect the carboxyl groups of residues Glu908 and Glu918; residues that form the water pore are shown as sticks, and water molecules are shown in blue as a ball-and-stick representation.

Partial hydration of the C-terminal pore agrees with crystallography data [18], but we did not identify hydrogen-bonded water wires connecting residue Glu309 and Glu318 in the nanosecond-long simulation. Water wires are proton-transport devices in biological systems [21], thus their presence is essential to infer whether this pore may serve as a proton-transporting conduit. Therefore, we used the structure at the end of the 300 ns trajectory to perform a 1.8 µs MD simulation of the protonated E2 state of the pump to establish the formation of water wires along the C-terminal proton release pathway. We found that the pore is partially hydrated during the first 0.63 µs of simulation time (Figure 3a) and contains on average four molecules of water during this time frame. The number of water molecules in the pore increases to an average of 7–11 for the remainder of the trajectory (Figure 3a). During the first 0.73 µs of simulation time, we did not detect the formation of water wires (Figure 3b); however, after t = 0.73 µs, we found ~130 stable (lifetime of 150–250 ps) hydrogen-bonded water wires that directly connect residues Glu908 and Glu318 (Figure 3c). The water wires identified in this pore are predominantly formed by six water molecules (Figure 3d); however, we also detected the formation of water wires composed of seven molecules of water in the time scales used here (Figure 3e). In all cases, the water wires originate from the carboxyl group of Glu908 and are stabilized along the pore by the hydroxymethyl group of Ser767, the carboxamide group of N911, the guanidine group of Arg836, and the hydroxyl group of Tyr377 (Figure 3d,e). The overall structure and stability of the hydrogen-bonded water wires are consistent with proton transport functionality [22], thus supporting the hypothesis that the C-terminal pore in SERCA serves as a conduit for proton release.

**Figure 4:**
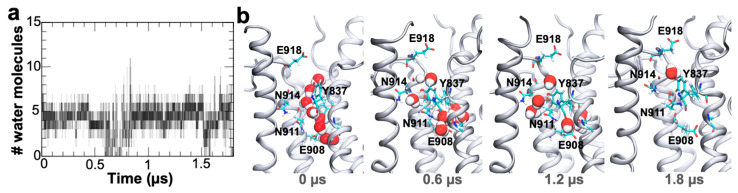
Identification of water wire formation in the C-terminal pathway following deprotonation of residue Glu908 in the E2 state of SERCA. (**a**) The number of water molecules found in the C-terminal pore throughout the 1.8 µs. molecular dynamics (MD) simulation of SERCA with residues Glu908 and Glu309 modeled as ionized. The number of water molecules was computed at 1 ns intervals. (**b**) Configuration of water molecules in the C-terminal pathway of SERCA following metal ion–proton release in the transport sites; the structures capture the disruption of the initially formed water wire and the absence of subsequent water wire formation events. SERCA structure is shown as ribbons, residues that form the water pore are shown as sticks, and water molecules in the C-terminal pathway are shown as spheres.

After establishing that the C-terminal pathway of protonated E2 state of SERCA is intrinsically hydrated and serves as a proton shuttling conduit element, we next determined whether proton release triggers gate closing in this pathway. This is especially important because residues Glu908 and Glu918 are present at the respective ends of the water wires; these residues are both a proton donor and acceptor, which may lead to proton reuptake from the cytosol back into the transport sites, which in turn may create a charge imbalance in the transport sites and interfere with the structural transitions necessary for SERCA activation [23]. Since explicit modeling of proton transport is not possible using conventional MD simulations, we simulated the effect of proton release using the structure of the fully hydrated C-terminal pore but with Glu908 modeled as ionized to mimic proton release from this residue; a detailed explanation of the modeling strategy is presented in the Materials and Methods section. Analysis of the trajectory showed that the C-terminal pathway is only partially solvated with 4–5 water molecules during the entire simulation time (Figure 4a), although complete pore dewetting was also detected in the trajectory (i.e., at 0.7–0.85 and 1.7 µs, Figure 4a). A closer inspection of the pore revealed that although water molecules occupy both ends of the C-terminal pathway, we do not detect the formation of continuous water wires connecting residues Glu908 and Glu918 throughout the 1.8 µs of simulation time (Figure 4b). This finding indicates that metal ion–proton exchange induces closing of the C-terminal proton release pore, effectually preventing proton exchange across this pathway. More importantly, the C-terminal pore does not undergo closed-to-open structural transitions in the timescales used here, indicating that proton recapture from the cytosol is unlikely to occur upon metal ion–proton exchange in the transport sites. These findings are consistent with the notion that the C-terminal proton release pathway is exclusive to the metal ion-free, protonated E2 state of the pump [18].

**Figure 5:**
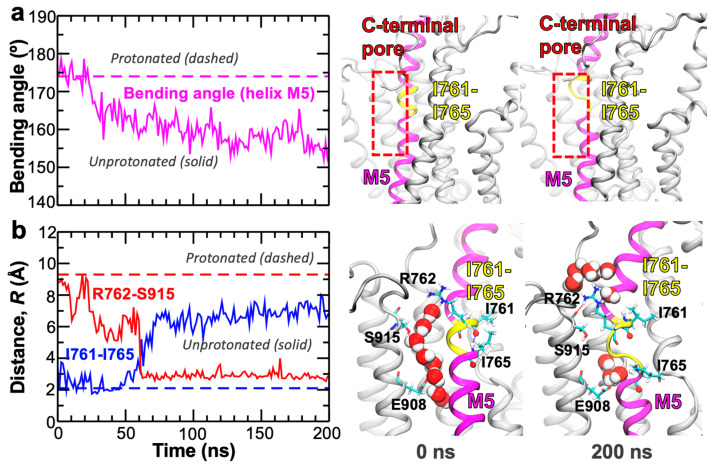
Local structural changes of SERCA inducing pore closure of the C-terminal pathway upon proton release from the transport sites. (**a**) Left panel: Time evolution of the bending angle of the transmembrane helix M5 of SERCA during the first 200 ns of in the 1.8 µs trajectory of the unprotonated E2 state SERCA; this timeframe captures the rapid bending of this helix in the trajectory. The dashed line shows the average bending angle calculated using the last 300 ns in the 1.8 µs trajectory of the protonated E2 state. Right panel: Ribbon representation of the rapid bending of transmembrane helix M5 (magenta) at residues Ile761–Ile765 (yellow). (**b**) Left panel: Time evolution of the distances Arg762–Arg915 and Ile761–Ile765 during the first 200 ns in the MD trajectory of unprotonated SERCA; this timeframe captures the concomitant interaction of Arg762–Ser915 and the breaking of the backbone hydrogen bond between Ile761 and Ile765. The dashed line shows the average distances calculated using the last 300 ns in the 1.8 µs trajectory of the protonated E2 state. Right panel: Structural representation for rapid closing of the C-terminal proton release pathway and inhibition of water wire formation induced by metal ion–proton exchange. SERCA is shown as ribbons, key residues involved in the closing of the pore are shown as sticks, and water molecules are shown as spheres.

**Figure A1:**
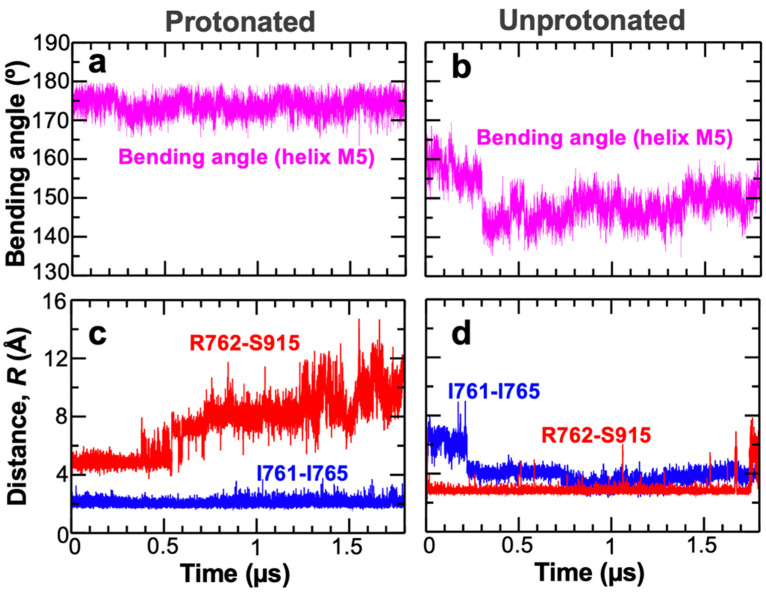
**(a**) and (**b**) time evolution of the bending angle of the transmembrane helix M5 of SERCA in the protonated and unprotonated E2 state of SERCA calculated from the 1.8 µs trajectories of the pump. (**c**) and (d) Table 762. Arg915 and Ile761–Ile765 in the full-length trajectory of protonated and unprotonated E2 SERCA.

Finally, we ask what structural mechanism drives the closing of the C-terminal proton release pathway of SERCA once proton transport has occurred through this pore. We focus on local structural changes occurring in the C-terminal pore that can lead to exclusion of water molecules from this region of the protein. Analysis of the trajectory revealed that deprotonation of Glu908 induces bending of the transmembrane helix M5 by 15–30° in the nanosecond timescale (Figure 5a and Figure A1, Appendix A). Bending of this transmembrane helix occurs within a region formed by residues Ile761-Ile765 (Figure 5b); the structural malleability of this region of SERCA is in agreement with NMR experiments showing that transmembrane helix M5 is prone to hinge-bending near the transport sites [24]. We found that bending of helix M5 is prompted and stabilized by the favorable interaction between the guanidine group of Arg762 and the backbone oxygen of Ser915 (Figure 5b and Appendix A). Indeed, the formation of this interaction occurs concomitantly with the breaking of the O(*i*)→N–H(*i*+4) bond between residues Ile761 and Ile765 early in the MD trajectory (Figure 5). These structural changes and interactions, which are not detectable in the fully hydrated C-terminal pathway of protonated E2 state of the pump (See Figure A1, Appendix A), are coupled to the rapid dissociation of the water wires and closing of the pore (Figure 5b). Once these structural transitions take place, the pore remains closed for the remainder of the simulation time (Figure 4). We note that in the trajectory of the protonated E2 state, the separation of Arg762 and Ser915 becomes more prominent upon the formation of water wires in the C-terminal pore (See Figure A1, Appendix A); conversely, Arg762 and Ser915 are clustered together (*R* < 5 Å) in the crystal structure of the Ca^2+^-free E1 state of the pump that features a closed structure of the C-terminal pathway [20,25,26]. These findings suggest that Arg762 plays a direct role in modulating the open-closed transitions of the pore; this functional role is supported by studies showing that removal of the positive charge at this position alters Ca^2+^ binding to SERCA [27]. More importantly, our simulation demonstrates that protonation plays a fundamental role in controlling the open-closed dynamics of the C-terminal pore via bending of the transmembrane helix M5. This functional role is supported by mutagenesis studies showing that single amino acid replacement of residues Ile761–Ile765 by alanine, a residue with a high propensity for helix formation [28], affects Ca^2+^ transport activity and apparent Ca^2+^ affinity of SERCA [29,30].

## 3. Conclusions

We used atomistic simulations to test the crystallography-based hypothesis that a C-terminal pathway in SERCA serves as a conduit for proton release from the transport sites [18], and to elucidate the mechanism for the operation of this proton-transporting pathway. We used molecular dynamics simulations starting from a crystal structure of the pump in the absence of non-cognate ligands. These simulations conclusively showed that the C-terminal is intrinsically hydrated in the protonated E2 state of SERCA and that this pore allows for the formation of hydrogen-bonded water wires that connect the transport sites with the cytosol. These findings are consistent with the proton transport functionality of this pathway, thus supporting the notion that the C-terminal pore in SERCA serves as a conduit for proton release. We performed additional atomistic simulations to determine whether the C-terminal proton release pathway undergoes open-to-closed structural transitions upon deprotonation of the E2 state. These simulations showed that bending of transmembrane helix M5 and the interaction Arg762–Ser915, which occur only upon metal ion–proton exchange in the transport sites, create a physical barrier against full hydration of the pore, thus preventing the formation of proton-transporting water wires. Remarkably, our simulations revealed that upon metal ion–proton exchange, the C-terminal pore is incapable of forming proton-transporting water wires, thus effectually inhibiting proton exchange in the transport sites during the E2-to-E1 transition of the pump [17]. Our simulations provide hypotheses for the function of the C-terminal proton release pathway that can be tested by functional mutagenesis and electrical measurements. For example, additional information may be gained by measuring charge transfer using microsomal vesicles containing SERCA adsorbed on a solid supported membrane [31,32]. These experiments can also be complemented with multiscale reactive molecular dynamics to quantify the free energy profile and timescale of the proton transport across the C-terminal proton pathway [26]. In summary, the structural evidence presented in this study supports the notion that the C-terminal proton release pathway is a functional element of SERCA, and also provides a mechanistic model for its operation in the catalytic cycle of the pump.

## 4. Materials and Methods

### 4.1. Simulation of the Protonated E2 State of SERCA for 300 ns

We used the crystal structure 3w5c [20] as an initial structure to map the C-terminal proton release pathway in the protonated E2 state of SERCA. We used this crystal structure because of its high resolution (2.5 Å), and because it was determined in the absence of exogenous inhibitors and/or non-cognate ligands [20]. To recapitulate the structural features of the protonated E2 state of the pump, we modeled side chains of transport site residues Glu309, Glu771, and Glu908 as protonated, and Asp800 as unprotonated [11,33]. Additionally, we used PROPKA [34,35,36,37] to adjust the ionization state of all other titratable residues to closely match those within a physiological pH range (7.0–7.2) [38,39]. This structure was then inserted in a pre-equilibrated 13 × 13 nm lipid bilayer composed of palmitoyl-2-oleoyl-*sn*-glycerolphosphatidylcholine (POPC) and solvated using TIP3P water molecules. We added K^+^, and Cl^−^ ions to neutralize the electric charge of the system and to produce an [KCl] of approximately 100 mM. We used the CHARMM36 force field topologies and parameters to model the protein, lipid, water, and ions [40,41]. We used this structure to simulate the hydration of the C-terminal proton release pathway. Briefly, we used NAMD [42], with periodic boundary conditions [43], particle mesh Ewald [44,45], a non-bonded cutoff of 12 Å, and a 2–fs time step. The equilibrated system was simulated without restraints for 300 ns at constant pressure (1 atm) and temperature (310 K) using a Langevin thermostat and an anisotropic Langevin piston barostat.

### 4.2. Simulation of the Protonated E2 State to Establish the Presence of Water Wires in the C-Terminal Pathway

We performed a MD simulation for a total of 1.8 µs to study the formation of proton-transporting water wires in the C-terminal proton release pathway of SERCA. To this aim, we used the structure of the protonated E2 state of SERCA obtained at the end of the 300 ns MD simulation described in Section 4.1. This structure was used ‘as is’ for this simulation, but the initial velocities for all atoms were generated randomly to generate an independent MD trajectory of this system. We simulated this system for 1.8 µs using NAMD [42] and the CHARMM36 force field topologies and parameters [40,41].

### 4.3. Simulation of Proton Release from Residue Glu908 in the E2 State of SERCA

We performed a MD simulation for 1.8 µs to simulate the effects of deprotonation and metal ion–proton exchange on the operation of the C-terminal pathway. Since classical MD simulations are not capable to treat proton transport explicitly, we model proton release from Glu908 based on the following assumptions: (i) the gatekeeper residue Glu309 was modeled as unprotonated to reflect the opening of the N-terminal pathway [18,19,33]; (ii) The transport site residues Glu771 and Glu908 are modeled protonated and unprotonated, respectively; these protonation states capture the release of a proton from Glu908 only [26]; and (iii) We explicitly modeled the rapid binding of K^+^ ion in the transport sites to recapitulate both metal ion–proton exchange as well as the charge conservation induced by the departure of a single proton from the transport sites [46,47,48]. For this purpose, we used the structure of the protonated E2 state of SERCA at 0.73 µs extracted from the 1.8 µs trajectory performed as described in Section 4.2. We chose this structure because it captures both the fully hydrated C-terminal pore and the formation of a water wire connecting residues Glu908 and Glu918. The structure was used ‘as is’ for this simulation, but we added two additional K^+^ ions to neutralize the electric charge of the system produced by the removal of protons from residues Glu908 and Glu309. This system was simulated with no restraints for 1.8 µs using the same protocol described in Section 4.1.

## Data Availability

The datasets used and/or analyzed during the current study are available from the corresponding author on reasonable request.
